# Implications of the degree of saturation on the mechanical behaviour of a slow-moving landslide in the Three Gorges region, China

**DOI:** 10.1007/s10064-025-04237-8

**Published:** 2025-04-02

**Authors:** Miguel Cueva, Enrico Soranzo, Ahsan Saif, Shun Wang, Wei Wu

**Affiliations:** 1 Institute of Geotechnical Engineering, University of Natural Resources and Life Sciences, Vienna, Feistmantelstrasse 4, A-1180 Vienna, Austria; 2 State Key Laboratory of Water Resources and Hydropower Engineering Science, Wuhan University, 299 Bayi Road, Wuhan, 430072 China; 3 Institute of Engineering Risk and Disaster Prevention, School of Water Resources and Hydropower Engineering, Wuhan University, Wuhan, 430072 China

**Keywords:** Huangtupo landslide, Slow-moving landslide, Shear-zone soil, Soil suction, Partial saturation, Shear strength

## Abstract

Slow-moving landslides are typically characterised by pre-existing shear zones composed of thick, clay-rich, and mechanically weak soil layers that exhibit heightened sensitivity to changes in moisture content and hydrological conditions. These zones, often governed by variations in suction and degree of saturation, play a critical role in the stability and long-term behaviour of slow-moving landslides. In this study, we investigate the influence of the degree of saturation on the mechanical properties of shear-zone soils from a reactivated slow-moving landslide in the Three Gorges Reservoir area, China. A series of laboratory experiments, including consolidation, reversal direct shear, and ring-shear tests, were conducted on reconstituted shear-zone soil samples at varying degrees of saturation. The test results indicate that increasing the degree of saturation has a marked impact on the compressibility of the soils, with saturated samples exhibiting greater compressibility and unsaturated samples demonstrating reduced compressibility. Both shear tests indicate that higher saturation leads to a reduction in peak and residual shear strength, likely due to elevated pore water pressures and a decrease in inter-particle bonding forces. These insights emphasise the need to account for varying degrees of saturation when analysing the mechanical behaviour of slow-moving landslides, contributing to an improved understanding of their deformation patterns and failure mechanisms.

## Introduction

Landslides, a pervasive natural hazard worldwide, pose significant threats to infrastructure, human life, and economic stability. These gravity-driven mass movements of soil and rock can cause catastrophic and irreversible damage, often resulting in substantial economic losses and tragic casualties (Guzzetti et al. 1999; Agliardi et al. 2020; Lacroix et al. 2020). While rapid, catastrophic landslides garner significant attention, slow-moving landslides, characterised by gradual and often episodic displacements, present unique challenges (Ranalli et al. 2010; Zangerl et al. 2010; Handwerger et al. 2016). Their subtle and progressive nature makes them difficult to detect and monitor, potentially leading to delayed recognition of the hazard and escalating risks to infrastructure and communities (Cascini et al. 2009, 2014; Pánek and Klimeš 2016; Scoppettuolo et al. 2020). Furthermore, while often perceived as less dangerous, slow-moving landslides can transition into catastrophic failures with devastating consequences (Tang et al. 2019). The 1963 Vajont landslide in Italy, which involved the gradual sliding of a massive rock mass into a reservoir, ultimately triggering a catastrophic wave that claimed thousands of lives, serves as a stark reminder of the potential for catastrophic failure in slow-moving landslides (Nonveiller 1987; Kilburn and Petley 2003). More recently, the 2014 Oso landslide in Washington, USA, exemplified the devastating consequences of a slow-moving landslide transforming into a rapid flow, resulting in significant loss of life and property damage (Iverson et al. 2015; Aaron et al. 2017).

The Three Gorges Reservoir (TGR) region in China, with its steep and rugged topography, complex geological formations, and fluctuating hydrological conditions, is particularly susceptible to the occurrence of landslides (Tang et al. 2019; Wang et al. 2022a). The impoundment of the mighty Yangtze River, coupled with seasonal variations in rainfall patterns, and the ongoing consequences of climate change, has significantly altered the hydrogeological regime in this dynamic region. Furthermore, the slope soils in the reservoir region may experience cycles of drying and wetting as a result of changes in the reservoir water level, exhibiting both saturated and unsaturated characteristics (Lai et al. 2014). These changes have reactivated and triggered numerous large, ancient landslides, many of which now exhibit slow and episodic movement patterns (Wang et al. 2014, 2016; Tang et al. 2015b).

Several factors can highly influence the compressibility and shear strength within the shear zones of pre-existing and first-time failure slow-moving landslides, such as soil suction and degree of saturation (Lian et al. 2021; Yang and Vanapalli 2024). Soil suction, the negative pore-water pressure relative to atmospheric pressure, acts as a crucial factor influencing the strength and deformation characteristics of unsaturated soils, which are widespread in the TGR region (Lai et al. 2014; Hu et al. 2018). Soil suction represents the capillary forces within the soil that bind particles together and contribute to the overall stability of slopes. Higher suction levels generally correspond to greater shear strength and lower compressibility, as the soil particles are held together more tightly by these capillary forces (Leroueil 2001; Zhou et al. 2016; Fredlund 2017). On the other hand, the degree of saturation, which describes the proportion of pore spaces filled with water, directly influences the magnitude of soil suction (Leroueil 2001; Fredlund 2017). As rainfall infiltrates the soil, the degree of saturation increases, leading to a reduction in suction. This decrease in suction weakens the soil, potentially reducing its shear strength and increasing its susceptibility to deformation (Springman et al. 2003; Abd IA et al. 2020). Fluctuations in soil suction represent a primary driver for the initiation and continued displacement of slow-moving landslides (Cascini et al. 2014; Hu et al. 2018). Furthermore, changes in saturation can also impact the soil’s volume change behaviour, potentially leading to swelling or shrinkage (Wijaya et al. 2015; Fredlund 2020), which can further influence the stability of slopes in the TGR region. Therefore, developing a complete comprehension of the factors governing the stability and deformation behaviour of these slow-moving landslides is of critical importance. The findings of this research will contribute to a better understanding of the role of the degree of saturation in the stability of slow-moving landslides, ultimately aiding in the development of more effective risk management strategies for the TGR region.

The novel comprehensive examination of how varying degrees of saturation influence the mechanical behaviour of shear-zone soils in a slow-moving landslide in the TGR area of China provides new insights into the compressibility, shear strength and deformation characteristics of the soil under different saturation levels. This study highlights the critical role of saturation in the mechanical properties of slow-moving landslides, emphasizing the need to account for varying saturation levels when analysing landslide behaviour. It also encompasses a novel investigation of the complex interplay of factors (capillary action, air entrapment and changes in inter-particle forces) that influence soil behaviour, offering valuable contributions to a better understanding of the failure mechanisms in slow-moving landslides. To thoroughly investigate this, we employed a combined experimental approach, utilising consolidation, reversal direct shear (RDS), and ring-shear (RS) tests. Additionally, to establish the relationships among the gravimetric water content, saturation degree, and soil suction, the shrinkage curve (SC) and the soil water characteristic curve (SWCC) were determined. Finally, based on these experimental results, the mechanical response of shear-zone soil specimens under a range of varying saturation levels is discussed.

**Fig. 1:**
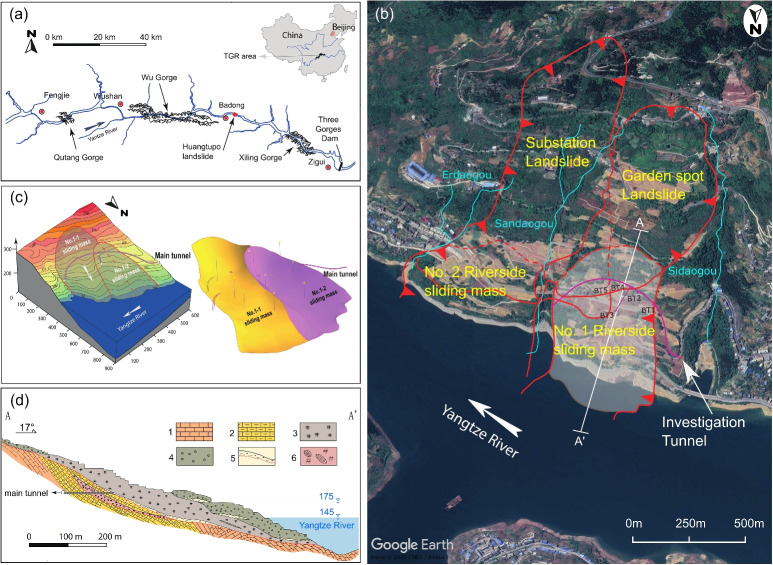
The Huangtupo landslide: (**a**) Location, (**b**) Plan view showing the multiple sliding masses with the investigation tunnel, (**c**) 3D topographic model and 3D double shear-surface model, (**d**) A-A’ cross-section through the No. 1 Riverside sliding mass: 1. limestone; 2. pelitic limestone; 3. dense soil and rock debris; 4. loose soil and rock debris; 5. shear zones with possible slip surfaces; 6. mudstone fraction zone. Modified after (Tang et al. 2015a; Wang et al. 2018a; Cui et al. 2021; Wang et al. 2022a)

## Background of the Huangtupo landslide

The Huangtupo landslide is a well-studied reactivated slow-moving landslide located in Badong County, Hubei Province, China, specifically on the south bank of the Yangtze River valley (Fig. 1a), within the geographic coordinates of $110^\circ 04' \text { to } 110^\circ 32'\, E \text { and } 30^\circ 28' \text { to } 31^\circ 28'\, N$, approximately 69 km east of the Three Gorges Dam. It is recognized as one of the largest and most hazardous landslides in the Three Gorges Reservoir region, covering an area of 1.35 km$^{2}$ and having a volume of approximately 70 million m$^{3}$ (Deng et al. 2000; Tang et al. 2019; Zou et al. 2020; Wang et al. 2022a). It has a crown elevation of approximately 600 m a.s.l., while its toe ranges from 50 to 90 m a.s.l. The toe of the landslide is submerged in the Yangtze River, with water levels fluctuating between 145 and 175 m due to the regulation of the Three Gorges Dam (Wang et al. 2014, 2016, 2021; Tang et al. 2015b). As a result of the water level fluctuations in the Three Gorges Reservoir, the original Badong County had to be relocated and reconstructed in the Huangtupo area from 1982 to 1991. However, subsequent investigations revealed that this area was affected by a large dormant landslide, prompting the relocation of the County to a site approximately 10 km west of Huangtupo (Deng et al. 2000; Hu et al. 2012a, b; Wang et al. 2014; Tang et al. 2015a; Gong et al. 2021).

According to a radiometric dating investigation by Tang et al. (2015a) the landslide formed over approximately 100,000 years, and consists of multiple sliding masses, as shown in Fig. 1b, divided by three gullies from east to west: Erdaogou, Sandaogou, and Sidaogou. The Sandaogou Valley divides the landslide’s surface into two groups, each containing two stacked sliding masses, with additional recent sliding masses emerging along the edges (Jiang et al. 2007; Chen et al. 2008; Hu et al. 2012a, b; Chai et al. 2013; Wang et al. 2014, 2016, 2018a). These include No. 1 Riverside sliding mass, No. 2 Riverside sliding mass, Substation Landslide, and Garden Spot Landslide. No. 1 Riverside sliding mass is located on the foreside and is adjacent to No. 2 Riverside sliding mass, while the Garden Spot landslide and Substation landslide overlay No. 1 Riverside sliding mass and No. 2 Riverside sliding mass, respectively. The No. 1 and No. 2 Riverside sliding masses at the front are adjacent to the Yangtze River and have their toes submerged in the river. Among the four landslides mentioned, No. 1 and No. 2 Riverside sliding masses are the most impacted by the fluctuations in the Three Gorges Reservoir and anthropogenic activities. Consequently, these two landslides exhibit higher deformation rates than the Substation Landslide and Garden Spot Landslide (Wang et al. 2018a; Cui et al. 2021). Additionally, as illustrated in Fig. 1c, the No. 1 sliding mass can be further divided into two partially overlapping secondary sliding masses, named No. 1-1 and No. 1-2 sliding masses (Wang et al. 2018a).

The Huangtupo landslide has been developed on dipping geological strata from the Middle Triassic Badong Formation (T$_{2}$b$^{2}$ and T$_{2}$b$^{3}$), which is characterised by an irregular alternation of mudstone, pelitic siltstone, argillaceous limestone, and limestone, including some weaker interlayers (Deng et al. 2000; Tang et al. 2015a; Wang et al. 2014). The general downslope dip of these strata and weak interlayers, typically ranging from 20$^{\circ }$ to 30$^{\circ }$ toward the Yangtze River, creates a geologic environment that is highly susceptible to slope instability and failure (Fig. 1d).

**Table 1:** Basic properties of the shear-zone soil

*W*(%)	$\rho$ (g/cm$^{3}$)	$\rho$_*d*_ (g/cm$^{3}$)	*G*_*s*_	*e*	*S*_*r*_(%)	*W*_*L*_(%)	*W*_*P*_(%)	*I*_*P*_(%)
13.5	2.15	1.89	2.69	0.42	85.8	32.48	17.27	15.21

*W* natural water content, $\rho$ bulk density, $\rho$_*d*_ dry density, *e* void ratio, *S*_*r*_ in-situ degree of saturation, ${W}_{L}$ liquid limit, ${W}_{P}$plastic limit, ${I}_{P}$ plasticity index

In 2012, the Three Gorges Research Centre for Geo-hazards constructed a 908-m-long main tunnel with five adits (BT1 to BT5) (Fig. 1b) that traversed the No. 1 Riverside sliding mass (Hu et al. 2012a, b; Tang et al. 2015a; Juang 2021). This infrastructure was built to investigate the distribution of the shear surface within the landslide and conduct in-situ tests (Tan et al. 2018; Wang et al. 2020). Furthermore, an extensive multifield, multisensor monitoring system was installed at the Badong Field Test Site to track surface and deep displacements (Wang et al. 2014; Tang et al. 2015b). Due to all the above-mentioned features e.g., large size, complex structure, and ongoing risk, the Huangtupo landslide has been the subject of extensive research and study making it a valuable case study for understanding the mechanisms of similar slides in the Three Gorges Reservoir Area. The landslide’s unique geological features, including its location, stratigraphy, and susceptibility to slope instability, have drawn the attention of engineers and researchers worldwide, who have conducted in-situ investigations and monitoring to better understand its dynamics and potential hazards (Deng et al. 2000; Cojean and Caï 2011; Liu et al. 2013; Tomás et al. 2014; Guardiani et al. 2024).

**Fig. 2:**
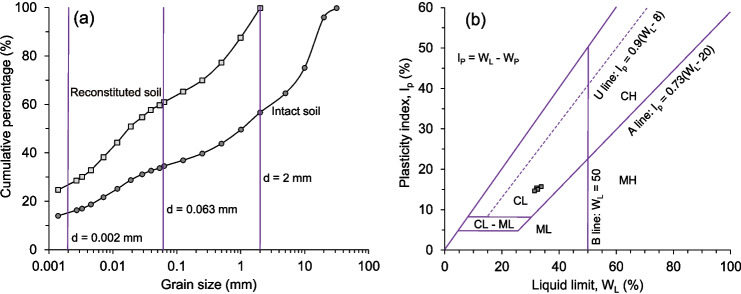
Physical properties of the shear-zone soil: (**a**) Grain size distribution, (**b**) Plasticity chart

## Materials and methods

### Test material

#### Preliminary tests and sample preparation

The soil samples investigated were obtained from the shear zone in Branch Tunnel #5 (BT5) inside the Riverside sliding mass I (Fig. 1b). These samples were analysed to determine the basic physical properties and grain-size distribution of the shear-zone material, as presented in Table 1. As can be seen in Fig. 2a, the shear-zone soil is predominantly composed of fine-grained soil with a significant fraction of gravel clasts, which are rounded to subangular in shape and range in diameters from 0.2 to 5 cm. According to the Casagrande Plasticity Chart shown in Fig. 2b, the reconstituted fine-grained portion of the soil samples can be classified as low-plasticity clay.

**Fig. 3:**
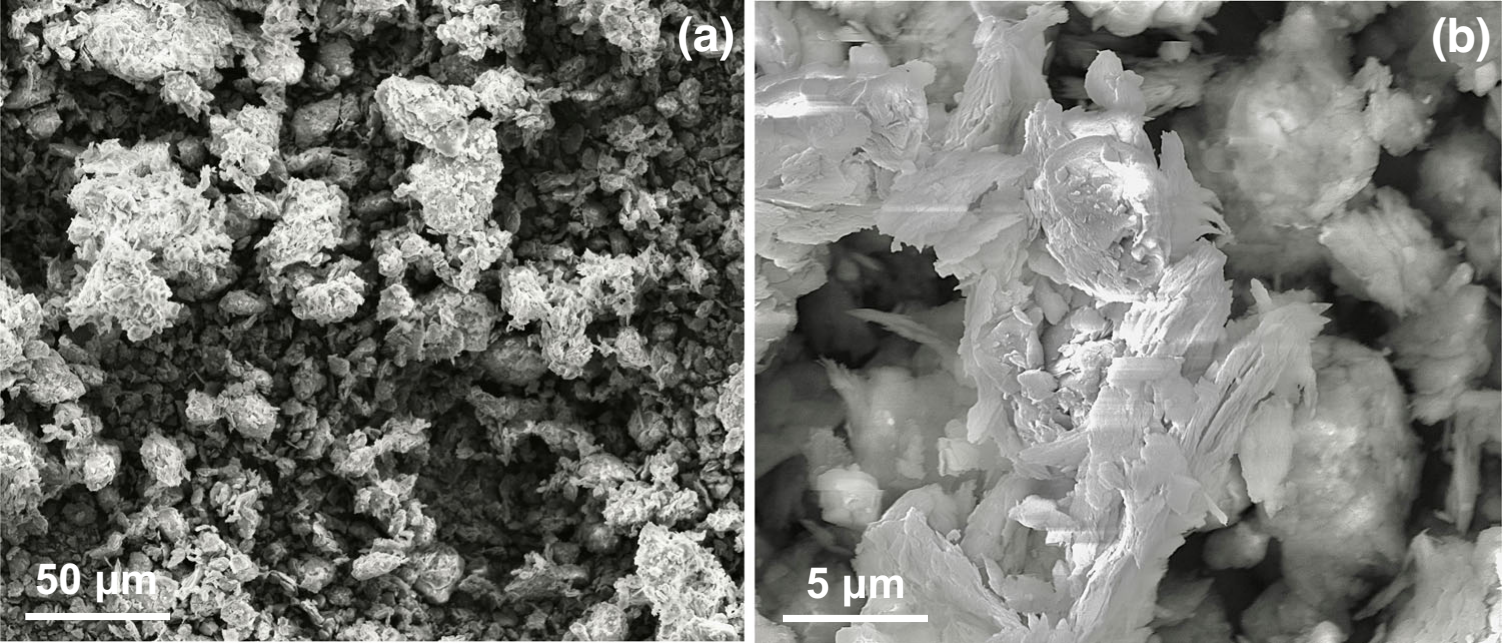
SEM images of the shear-zone soil: (**a**) x1000, (**b**) x10000

**Fig. 4:**
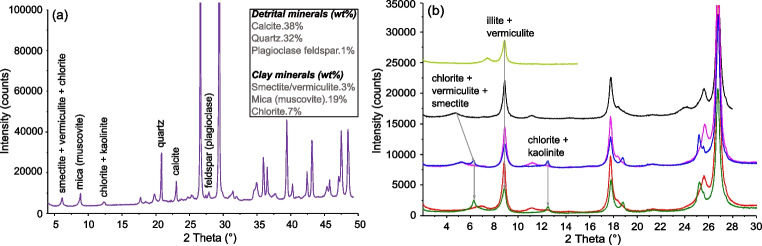
Mineralogical composition of the shear-zone soil: (**a**) Bulk mineral analysis, (**b**) Clay mineral analysis

Scanning electron microscopy (SEM) was utilised to analyse the soil fabric or microstructural characteristics of the shear-zone specimens. Figure 3 presents the scanning electron micrographs of the shear-zone samples obtained at various magnifications. Based on the micrographs, the surface of the samples appears to exhibit irregular, flaky sheet-like structures typical of clay minerals. The mineral boundaries are arranged in a mosaic pattern, and the pores within the samples are not readily apparent. X-ray diffraction (XRD) was conducted to determine the bulk mineral and clay mineral composition of the soil samples. The results indicate that the mineral composition of the shear-zone soil comprises approximately 71% detrital minerals and 29% clay minerals. The principal constituents of the soil samples are calcite, quartz, and the clay minerals illite and chlorite, as shown in Fig. 4.

To mitigate the influence of material heterogeneity within the intact shear-zone soil on the compression and shear strength parameters, which can lead to scattered results and the size effect in laboratory tests (Wang et al. 2020, 2022b; Cui et al. 2021), the following sample preparation procedure was employed. First, the shear-zone soil samples were air-dried and sieved to remove particles exceeding 2 mm in size. This step was taken to ensure a more homogeneous soil matrix for testing. Subsequently, the reconstituted soil was mixed with water to match the natural water content, and then the mixture was stored in a sealed container for more than a month. This extended storage period allowed for the uniform distribution of moisture throughout the soil sample, further reducing potential heterogeneity that could impact the test results.

#### Soil-water retention behaviour

The volume of certain soils is subject to change as their water content fluctuates. As a result, it is necessary to measure the soil’s shrinkage curve, which provides data on the relationship between volumetric changes, expressed in terms of void ratio, and gravimetric water content. Furthermore, the shrinkage curve is a prerequisite for constructing the soil water characteristic curve in terms of the degree of saturation (Fredlund et al. 2002a; Fredlund 2017).

**Fig. 5:**
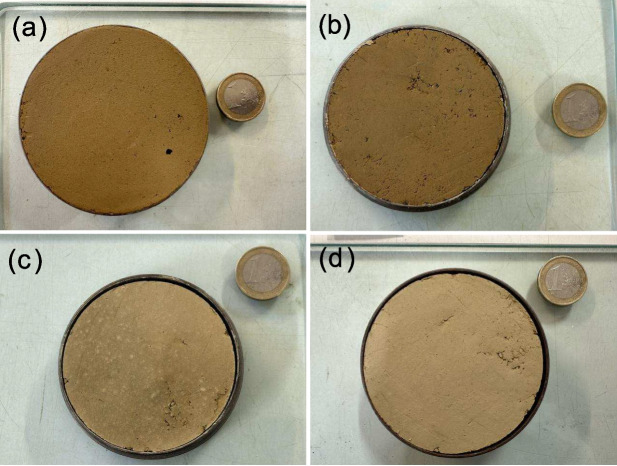
Soil specimen under different degrees of saturation: (**a**) 100%, (**b**) 78%, (**c**) 54%, (**d**) 28%

**Fig. 6:**
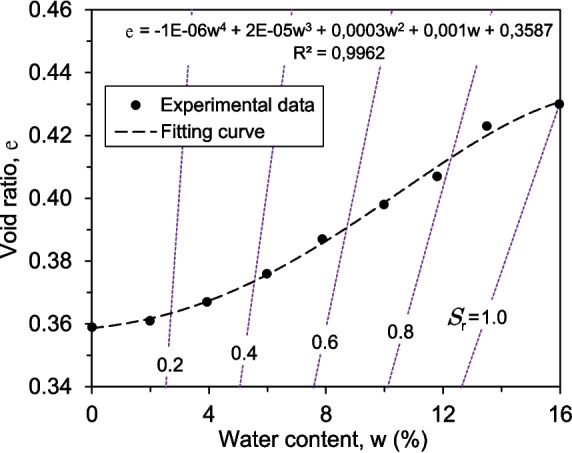
Schematic representation of the shrinkage curve (SC) of the shear-zone soil specimen

For this study, the shrinkage curve (SC) was determined using a non-destructive direct measurement method. The dimensions of the soil sample were measured using a Vernier calliper. This method requires assuming a homogeneous specimen geometry while drying to calculate the volume change. However, due to the realistic non-homogeneous geometry, multiple diameter and height measurements were taken and averaged to obtain a higher degree of confidence. This technique offers advantages such as simplicity, speed, and non-destructiveness of the specimen. Initially, the sample within the ring was submerged in water and allowed to fully saturate over more than 24 hours. The specimen diameter and height were 7.0 and 1.87 cm, respectively. Subsequently, once the sample reached a near-saturated state, a Vernier calliper was utilised to measure the volume changes at varying water contents as the sample dried. This non-destructive direct measurement approach allowed us to monitor the volume alterations during the drying procedure (Fig. 5), generating valuable data to construct the shrinkage curve, as shown in Fig. 6. The shrinkage curve illustrates the relationship between the void ratio (e) and the water content (w) of a soil specimen during the drying process. As expected, the void ratio decreased steadily as the water content reduced, indicating an overall shrinkage of the specimen. This behaviour is consistent with the typical response of soils to drying, where the loss of water leads to a reduction in the volume of the soil pores, thereby decreasing the void ratio. The experimental data points are closely aligned with the fitting curve, where the high coefficient of determination (0.99) indicates an excellent fit between the experimental data and the model, suggesting that the polynomial equation accurately describes the relationship between void ratio and water content for this soil specimen. The degree of shrinkage observed in the specimen was not particularly significant. This can be attributed to the relatively high sand content in the soil sample. Sandy soils typically exhibit less pronounced volume changes with variations in moisture content compared to clayey soils. Additionally, the absence of expansive clay minerals, which are known to undergo significant volume changes with moisture variations, further explains the modest shrinkage observed. Moreover, Fig. 6 includes reference lines representing different degrees of saturation, which enhance the correlation between the void ratio, water content, and degree of saturation, providing a more comprehensive understanding of the soil’s behaviour. The measurements were periodically recorded to precisely capture the volume change behaviour.

**Fig. 7:**
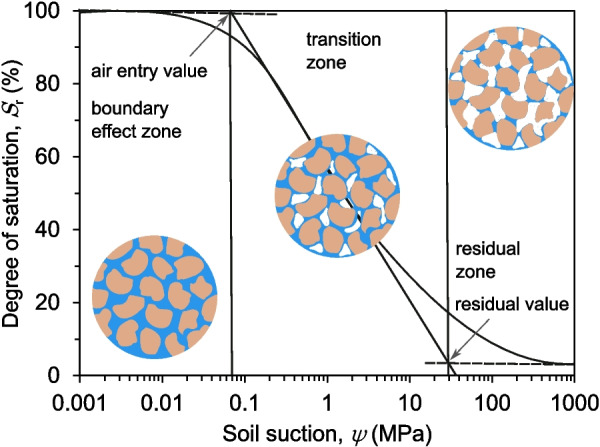
Schematic illustration of a typical soil water characteristic curve (SWCC) showing the different zones of unsaturation

The soil-water characteristic curve (SWCC) is crucial for understanding unsaturated soil behaviour. As shown in Fig. 7, this curve typically exhibits three distinct stages: a boundary effect stage, a transition stage, and a residual stage of unsaturation. These stages are conventionally delineated by two key parameters - the air-entry value and the residual value, respectively. The air-entry value represents the suction level at which air begins to enter the soil pores, while the residual value denotes the water content that remains in the soil even at very high suction levels (Fredlund 1994; Fredlund et al. 2002b). However, these parameters highly depend on the shrinkage behaviour of the soil used. In non-shrinking soils such as sand, they remain consistent regardless of whether the SWCC is based on gravimetric water content, volumetric water content, or degree of saturation. Conversely, for shrinking soils like expansive clays, they vary depending on the specific type of SWCC used (Fredlund et al. 2011; Wijaya et al. 2015; Fredlund 2020). Employing a SWCC based on gravimetric water content for shrinking soils will result in assuming fixed air-entry and residual values and neglecting the volume change effects. To accurately determine the air-entry value and the residual value in such cases, it is recommended to use the SWCC based on the degree of saturation (SWCC-S) instead. The SWCC-S can be generated by integrating data from the SWCC based on gravimetric water content with the soil’s shrinkage curve (SC), which provides the void ratio and water content data necessary to calculate the degree of saturation and transform the SWCC-w into SWCC-S (Fredlund 2002; Fredlund et al. 2002a; Wijaya et al. 2015; Fredlund 2017).

The SWCC of the shear-zone soil specimens was determined by employing the Pressure Plate Method (PPM) with suctions ranging from 0.01 MPa to 1.5 MPa. For the PPM tests, the cylindrical soil specimens were first placed in a partially water-filled plastic container for over a week to achieve saturated conditions. A pressure plate apparatus with a maximum 1.5 MPa high air entry ceramic porous plate was then utilised. The high air entry plate was initially saturated with de-aired water before being sealed inside the pressure plate chamber. The soil specimens were then positioned on the porous plate, which was connected to a small pipe to discharge any excess water into an external plastic bottle. The pressure chamber was securely closed and sealed, and air pressure was thoroughly applied. After each air pressure increment, some water flowed from the sample through the pipe into the plastic bottle. Once the flow became negligible, typically after a few days or weeks, indicating the sample had reached hydraulic equilibrium for the specific applied pressure, the air pressure was reduced to zero. The soil specimens were then removed from the pressure plate chamber, and their weights were measured to determine the corresponding water content. This procedure was repeated incrementally for the subsequent air pressure levels. The axis translation technique was employed to ascertain the suction in the pressure plate. This involved increasing the air pressure while maintaining the pore water pressure at atmospheric pressure, following the definition of matric suction:

1 $$\begin{aligned} \psi = u_a - u_w \end{aligned}$$

with $u_a$ and $u_w$ being the pore air and pore water pressures, respectively.

**Fig. 8:**
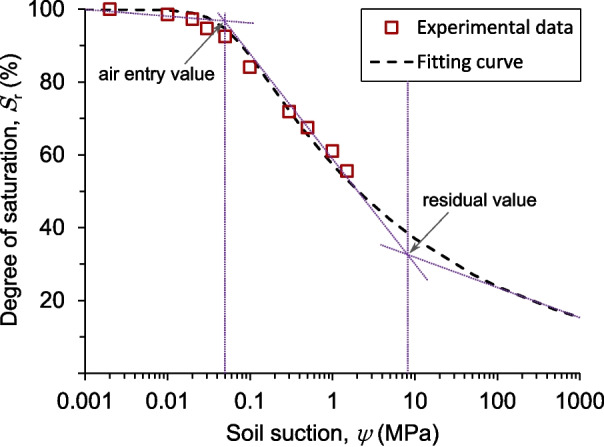
Soil water characteristic curve (SWCC) along the drying branch, obtained through the Pressure Plate Method (PPM). Best-fit curve to the experimental data by using the van Genuchten (1980) empirical model

The SWCC obtained through the PPM for the drying branch is presented in Fig. 8. It depicts the soil’s moisture content as it transitions from saturated to nearly dry conditions. For the shear zone soil tested, an air-entry value of approximately 0.05 MPa and an estimated residual value of 8.5 MPa were determined. The empirical Van Genuchten model (van Genuchten 1980), a two-parameter model, was employed to fit the experimental data. The Van Genuchten model, expressed in terms of the degree of saturation for convenience, considers the residual saturation degree $S_r$ as follows:

2 $$\begin{aligned} S = S_r + \frac{1 - S_r}{[1 + (\alpha \psi )^n]^ \frac{n - 1}{n}} \end{aligned}$$

where the parameters $\alpha$ and *n* are empirical constants, with $\alpha$ being the reciprocal of the air-entry value. This is an extrapolation based on the van Genuchten Eq. 2. The equation was fitted by minimising the Mean Squared Error between the ground truth and the predictions. Given that no observations for suction values larger than 1.5 MPa exist, the extrapolation for such values must be taken carefully. As expected, the decrease in saturation with increasing matric suction occurs gradually and slowly due to the high density and fine-grained nature of the shear-zone soil.

### Test apparatus, test plan and procedure

**Fig. 9:**
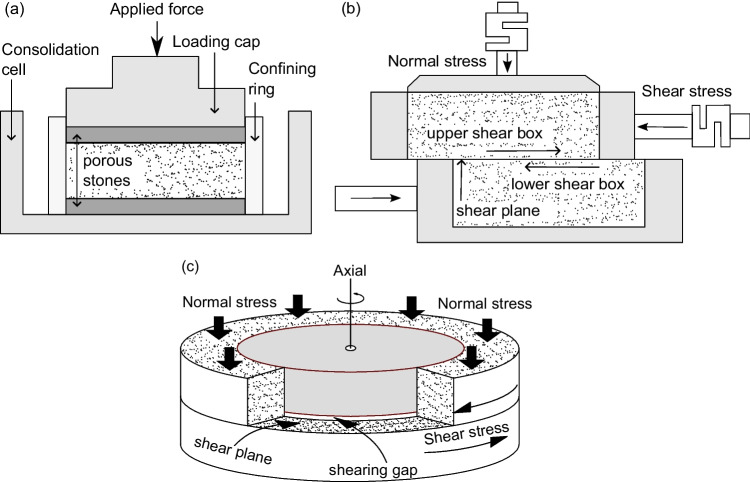
Tests apparatus: (**a**) Oedometer, (**b**) Direct shear, (**c**) Ring shear. Modified after (Wang et al. 2018b)

An oedometer apparatus was used to evaluate the compressibility, consolidation, and swelling properties of the shear-zone soil samples. The testing setup includes a metal frame, a lever arm system for applying incremental loads, a dial gauge for precise measurement of sample deformation, and a consolidation cell to securely hold the samples during testing. The consolidation cell consists of two porous stones, a metal confining ring, and a loading cap (Fig. 9a). To assess the shear strength parameters in both peak and residual states, two testing methods were used: reversal direct shear tests and ring shear tests. The in-house developed RDS apparatus comprises a reaction metal frame, hydraulic jacks, and a shear box with ring-shaped parts, as depicted in Fig. 9b. The Wille Geotechnik ring shear apparatus includes a loading system, a data acquisition system, and a shear box. Vertical stress and torque are managed by a servo-actuated loading piston and a servo-hydraulic motor, respectively, and a transducer measures vertical displacement during testing. As shown in Fig. 9c, the shear box accommodates a ring-shaped sample, which is confined by upper and lower rings separated by a shearing gap. During shearing, ribs on the porous ring plates transfer torque to the sample, forming the shear surface.

**Table 2:** Test programme of the consolidation tests

*S*_*r*_(%)	Loading stage (kPa)	Unloading stage (kPa)	Reloading stage (kPa)
100	25, 50, 100, 200, 400, 800	400, 200, 100, 50	100, 200, 400, 800, 1600
78	25, 50, 100, 200, 400, 800	400, 200, 100, 50	100, 200, 400, 800, 1600
54	25, 50, 100, 200, 400, 800	400, 200, 100, 50	100, 200, 400, 800, 1600
28	25, 50, 100, 200, 400, 800	400, 200, 100, 50	100, 200, 400, 800, 1600

The consolidation and shear tests were conducted under both saturated and partially saturated conditions to investigate the influence of the degree of saturation on the mechanical behaviour of the shear-zone soil. The unsaturated tests were performed at degrees of saturation of 78%, 54%, and 28%. Before conducting the tests, the general physical properties of the soil samples were measured as presented in Table 1. Using this data, the initial void ratio and degree of saturation under natural conditions were calculated. However, due to volume changes, particularly the shrinkage of the samples under different degrees of saturation (Fig. 5), a relationship between void ratio, water content, and degree of saturation was established (Fig. 6).

The consolidation tests involved placing the samples within the internal ring of the holder and subjecting them to incremental loading, unloading, and reloading phases, as detailed in Table 2. A dial gauge was used to measure the deformation over time, providing insights into how the soil consolidates under pressure and swells during unloading. For the saturated test, the consolidation cell was filled with water, and the sample was allowed to saturate for at least 24 hours before incrementally applying each loading step. The unloading sequence reversed the loading process, and a reloading sequence, identical to the initial loading, was conducted to complete the test.

The RDS test procedure involved gradually pulling the shear box back to its original position after each shearing phase. This method measures residual shear strength through large shear displacements (Di Maio et al. 2013; Kang et al. 2020, 2022b; Zhou et al. 2023). The tests were conducted under four normal stress levels (50, 100, 200, and 400 kPa) using a low shear displacement rate of 0.02 mm/min to maintain drained conditions. The saturated tests involved flooding the shear boxes with water, allowing 24 hours for saturation and an additional 24 hours for primary consolidation. The samples were reconsolidated for 30 minutes before each shearing, and four cycles of 10 mm each were performed to achieve the residual state. Shear displacements for the four sets of curves were recorded from 0, 10, 20, and 30 mm, ensuring a comprehensive analysis of the displacement behaviour. A summary of the test programme is provided in Table 3. The RS tests, in contrast to the RDS tests, allow for large shear displacements without reversing the shear direction (Tika et al. 1996; Wang et al. 2010; Scaringi and Di Maio 2016; Scaringi et al. 2018; Hu et al. 2018; Kang et al. 2022a; Duque et al. 2023). The saturation and consolidation process for the saturated specimens was similar to the RDS tests. The soil samples were consolidated under normal effective stresses of 100, 200, and 400 kPa and then sheared at a slow rate of 0.02 mm/min until the residual strength was reached, to prevent pore pressure increase. The test programme details are presented in Table 3.

**Table 3:** Test programme of the shearing tests

Shearing test	${S}_{r}$(%)	${\sigma }_{n}$(kPa)	$\dot{\gamma }$(mm/min)
RDS	100, 78, 54, 28	50, 100, 200, 400	0.02
RS	100, 78, 54, 28	100, 200, 400	0.02

${S}_{r}$ degree of saturation, ${\sigma }_{n}$ normal stress, $\dot{\gamma }$ shear rate

**Fig. 10:**
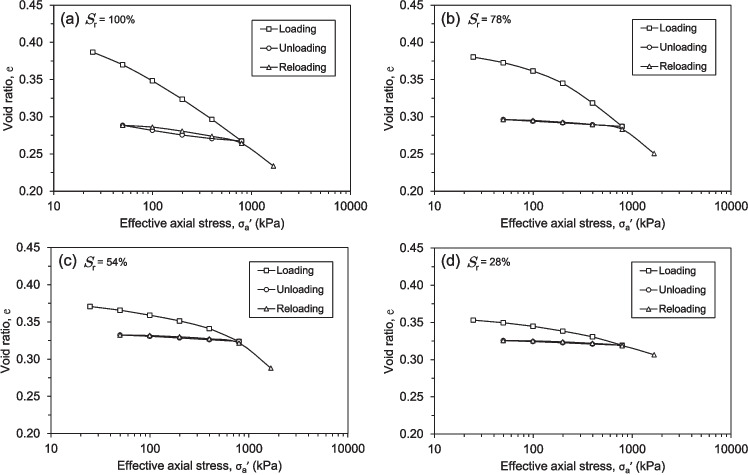
Compression and swelling behaviour of the soil specimens obtained from the shear zone under varying degrees of saturation: (**a**) 100%, (**b**) 78%, (**c**) 54%, (**d**) 28%

## Results

### Compressive behaviour

The results of the consolidation tests were obtained on soil specimens with a wide range of initial water contents, void ratios and corresponding degrees of saturation, ranging from 28% to 100% as shown in Fig. 10. It can be clearly observed that the initial void ratio of the soil samples is directly dependent on their initial water content. This effect is attributed to the shrinkage behaviour exhibited by the soil samples during the drying process. As the water content decreased, the soil specimens underwent volumetric changes, leading to a reduction in void ratio that was proportional to the initial moisture conditions.

The test results demonstrated a broadly consistent pattern across varying water contents and loading, unloading, and reloading conditions. During the initial loading stage, the fully saturated soil sample exhibited the greatest compressibility, with a void ratio reduction of approximately 0.120. This was followed by the unsaturated soil samples, which displayed void ratio reductions of 0.090, 0.047, and 0.034 for degrees of saturation of 78%, 54%, and 28%, respectively. For the unloading phase, the unsaturated specimens exhibited a remarkably almost similar and negligible swelling response compared to the fully saturated sample, which demonstrated a dilation nearly triple in magnitude. This trend can be clearly observed in the near-horizontal slopes of the swelling curves depicted in Figs. 10b, c, and d. For the reloading stage, the decrease in void ratio precisely mirrored the expansion during the preceding unloading phase. In other words, the consolidation curves for the partially saturated samples directly traced the swelling curves in reverse. The sole exception was the fully saturated sample, whose reloading curve deviated from the unloading curve, forming a distinct hysteresis loop, as illustrated in Fig. 10a.

**Fig. 11:**
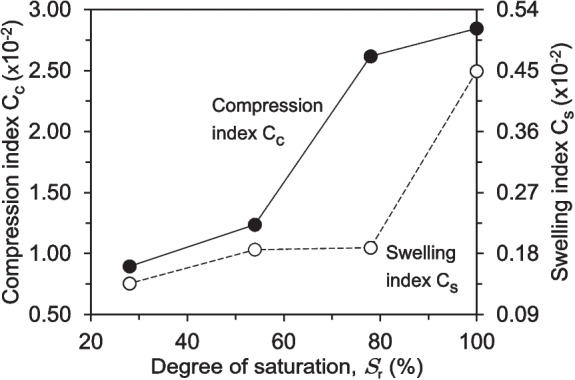
Variation of compression index and swelling index of the soil specimens under varying degrees of saturation

The preceding data on the loading and unloading phases enabled the determination of the variation in the compression index, $C_c$, and the swelling index, $C_s$, as a function of the degree of saturation, as shown in Fig. 11. The compression index was derived from the virgin consolidation curve, reflecting the soil’s response to increasing vertical stress during the first loading cycle, while the swelling index was determined from the unloading curve, capturing the soil’s elastic rebound upon stress reduction. To determine the $C_c$, the slope of the compression curve was taken within the stress range of 800 kPa to 100 kPa, a range selected to capture the primary consolidation behaviour where the most significant reduction in void ratio occurs. Similarly, the $C_s$ was obtained from the unloading curve over a stress range of 400 kPa to 100 kPa, representing the soil’s ability to recover volume upon unloading. This procedure was applied to all degrees of saturation to generate Fig. 11, highlighting the evolution of compressibility and swelling characteristics across different saturation levels.

**Fig. 12:**
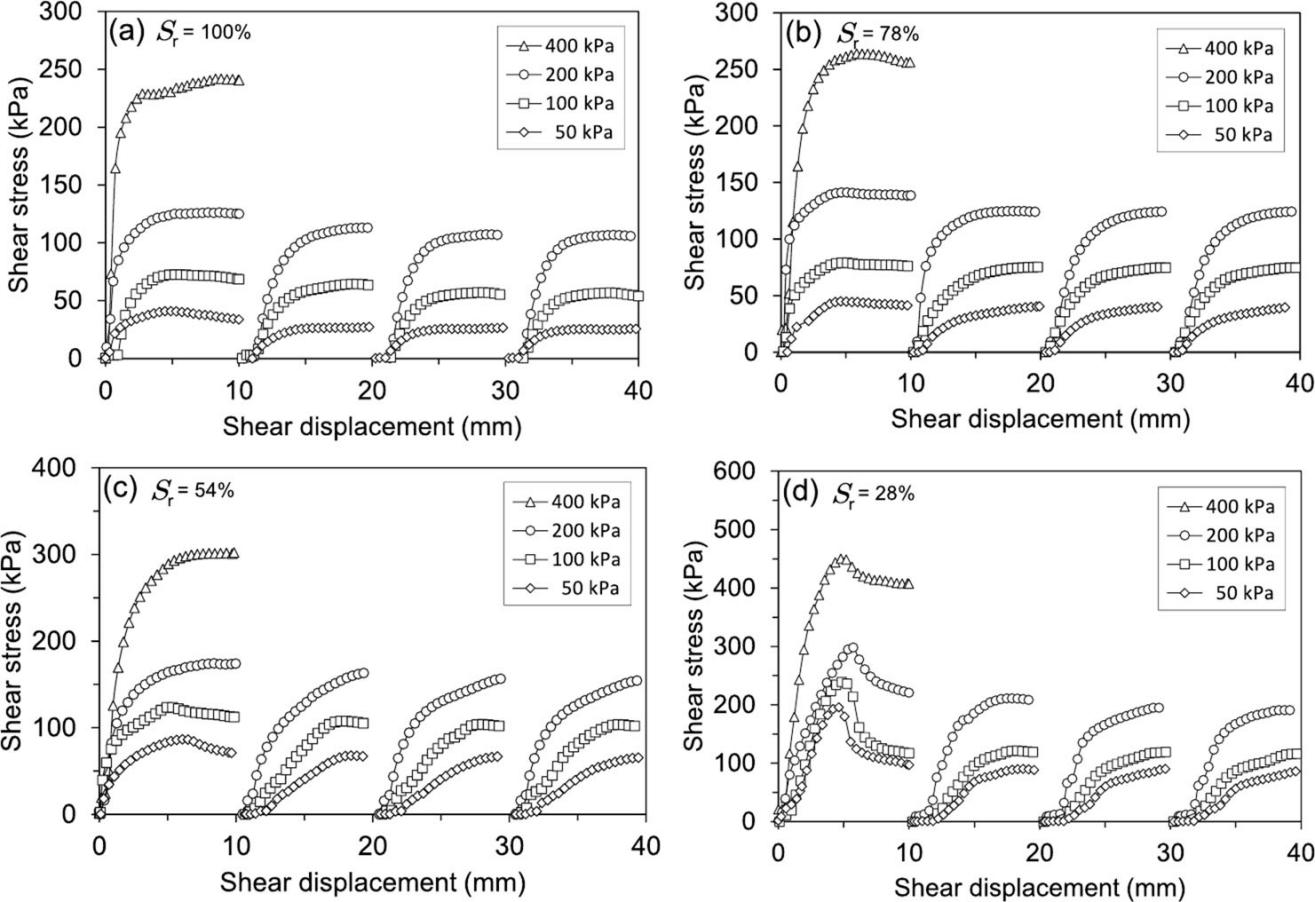
Shear stress-shear displacement curves of soil specimens subjected to varying effective normal stresses obtained from RDS tests: (**a**) 100%, (**b**) 78%, (**c**) 54%, (**d**) 28%

The highest compression index was observed in the fully saturated sample, followed by a relatively small decrease for the sample with a 78% degree of saturation. However, the compression index presented a significant drop from 0.02617 to 0.01237 for the 78% and 54% degrees of saturation, respectively. Regarding the swelling index, the most pronounced change occurred between the soil samples with 100% and 78% degrees of saturation, decreasing from 0.00449 to 0.00189. The samples with 78% and 54% saturation exhibited an almost identical swelling index, but the swelling index decreased further for the sample with a 28% degree of saturation.

### Shear strength behaviour

#### Reversal direct shear tests

The shear stress-shear displacement behaviour of saturated and partially saturated soil specimens under varying effective normal stresses is depicted in Fig. 12. Four consecutive shearing cycles of 10 mm each from 0, 10, 20, and 30 mm were required to reach the residual state. The soil sample with the lowest degree of saturation (Fig. 12d) displayed peak and residual strengths nearly double that of the fully saturated sample (Fig. 12a). Compared to the fully saturated sample, the peak and residual strengths increased for the soil specimen with a 78% degree of saturation (Fig. 12b). A similar trend was observed for the 54% degree of saturation sample (Fig. 12c), which also displayed increased peak and residual strengths compared to both the 78% degree of saturation sample and the fully saturated condition. Irrespective of the degree of saturation, all soil samples experienced a significant reduction in shear strength after undergoing the four shearing stages. Nevertheless, the unsaturated sample with a 28% degree of saturation displayed a particularly dramatic decline in its peak strength following the initial shear cycle. This more pronounced loss of peak strength in the 28% degree of saturation sample, compared to the other degrees of saturation, underscores the critical influence of the degree of saturation on the soil’s mechanical behaviour and its resistance to shearing.

**Fig. 13:**
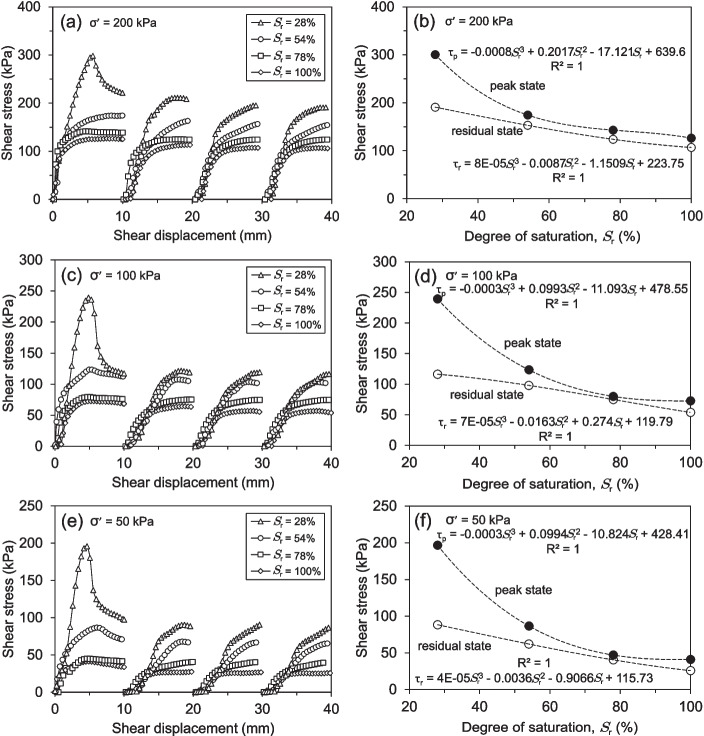
RDS test results sheared at different effective normal stresses: (**a, c, e**) Shear stress-displacement behaviour of soil specimens with varying initial degrees of saturation, (**b, d, f**) Variation of shear stresses as a function of the degree of saturation, highlighting the relationship between saturation levels and shear strength

Figure 13 presents the results of shear stress versus shear displacement for soil specimens with degrees of saturation ranging from 28% to 100%, tested under effective normal stresses of 200 kPa, 100 kPa, and 50 kPa and a displacement rate of 0.02 mm/min. As shown in Fig. 13a, the shear stress-shear displacement curves indicate that both peak and residual shear strengths decrease as the degree of saturation increases. The sample with the lowest degree of saturation (28%) exhibits a clear distinction between peak and residual shear strength, whereas the fully saturated sample and those with intermediate saturation levels (54% and 78%) display closer peak and residual values. This suggests that lower saturation levels contribute to a more pronounced peak strength before shearing reduces the sample to its residual state. The same trend is observed under different effective normal stresses, as depicted in Figs. 13c and e. Regardless of the applied normal stress, samples with higher degrees of saturation exhibit lower peak shear strengths, with the largest peak-to-residual strength drop occurring in the soil sample with a 28% degree of saturation. As the effective normal stress decreases from 200 kPa (Fig. 13a) to 100 kPa (Fig. 13c) and 50 kPa (Fig. 13e), the overall shear strength values decrease correspondingly. To further analyse the effects of the degree of saturation on peak and residual shear strengths, Fig. 13b, d, and f illustrate the variations in peak and residual shear strength as a function of the degree of saturation for each normal stress level. The results confirm a non-linear reduction in peak strength with increasing saturation, with the most significant drop occurring in the soil sample with a 28% degree of saturation. In contrast, residual shear strength follows a more gradual and almost linear decline across all normal stress levels. Additionally, the influence of effective normal stress and degree of saturation on peak strength is more pronounced at lower effective normal stresses and degrees of saturation, as seen in Figs. 13d and f. However, as saturation increases, the difference in peak strength across different normal stresses diminishes, indicating a saturation-dependent mechanical response in the soil.

**Fig. 14:**
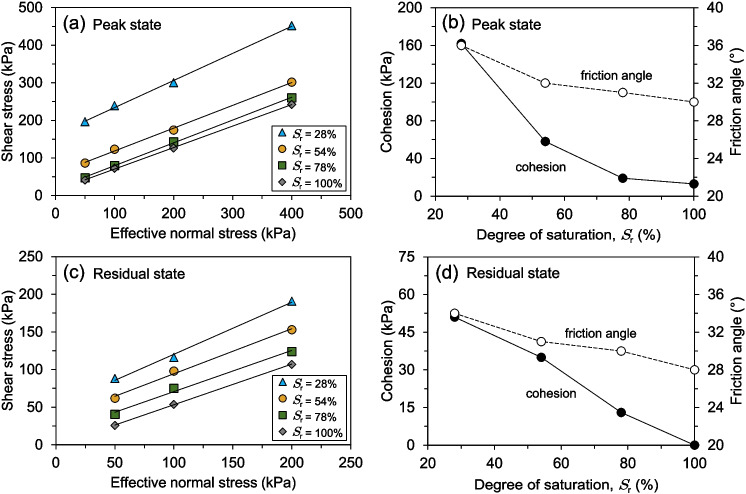
Shear strength envelopes and variation of cohesion and friction angle of soil specimens with different degrees of saturation from RDS tests: (**a, b**) Peak state, (**c, d**) Residual state

The shear strength envelopes for the peak and residual states under varying effective normal stresses and degrees of saturation are illustrated in Figs. 14a and c. A clear reduction in both peak and residual shear strength is observed as the degree of saturation increases. However, the decrease in peak strength is more pronounced than the reduction in residual strength. For the peak state, the most significant changes in strength occur at the highest degrees of saturation. Conversely, the decrease in residual shear strength exhibits a more continuous trend with an increase in the degree of saturation. It is observed that the soil samples with the highest and lowest shear strength values correspond to the lowest and highest degrees of saturation, respectively. The variation in cohesion and friction angle of the soil samples for both the peak and residual shear strength states under different degrees of saturation is shown in Figs. 14b and d. Both the peak and residual shear strength parameters decrease as the degree of saturation increases. For both shear strength states, the friction angle exhibits a nearly identical decreasing trend as the degree of saturation rises. However, the cohesion experiences a significant reduction, particularly when the degree of saturation increases from 28% to 54% in the peak state. In contrast, the cohesion remains almost constant for the 78% and 100% degrees of saturation. Regarding the residual state, the cohesion decreases in a nearly linear fashion as the degree of saturation increases.

**Fig. 15:**
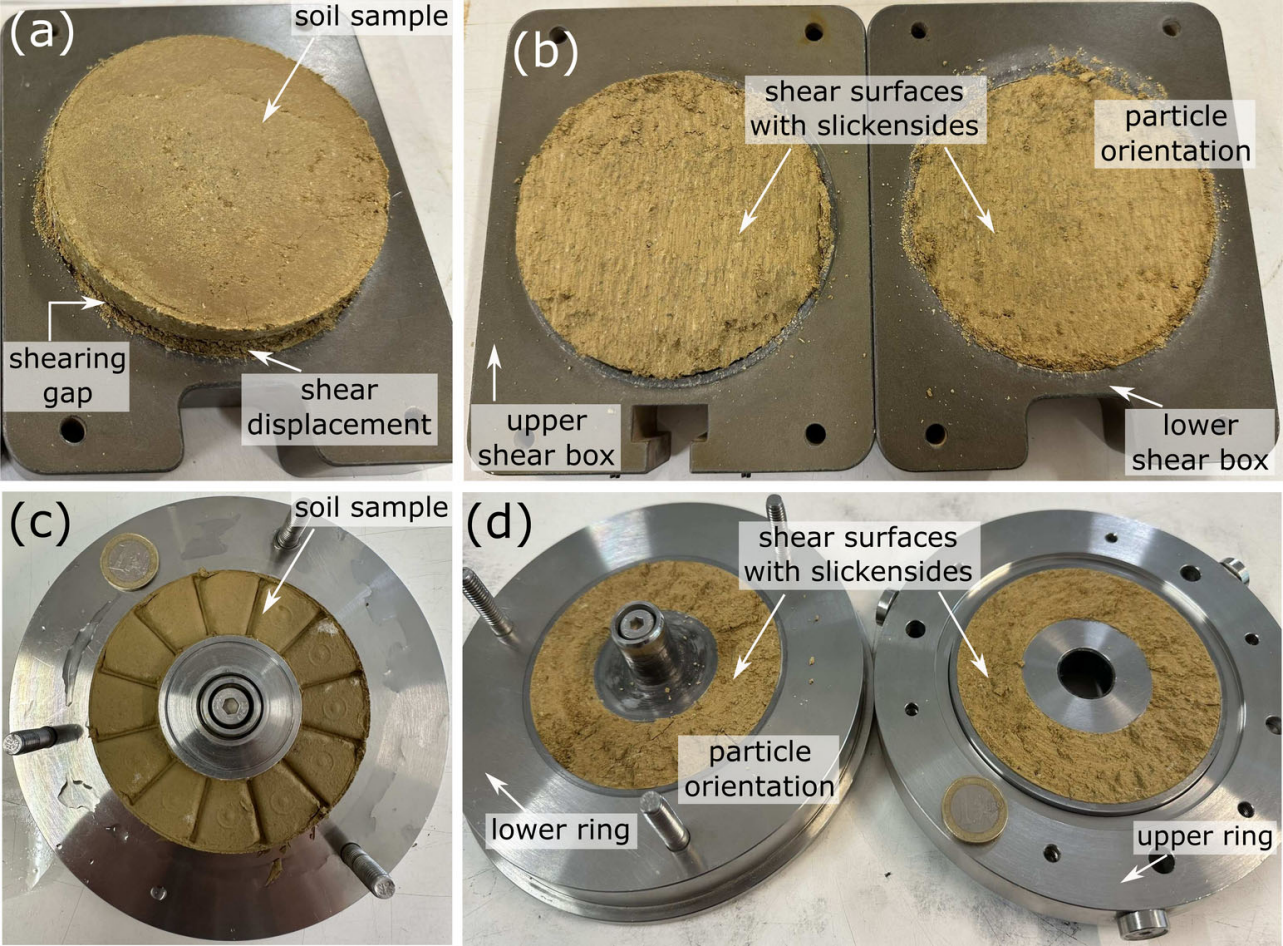
Photographs of shear-zone soil samples with a 28% degree of saturation after shearing: (**a, b**) RDS and (**c, d**) RS test specimens, showing shear zone morphology and post-shearing deformation

**Fig. 16:**
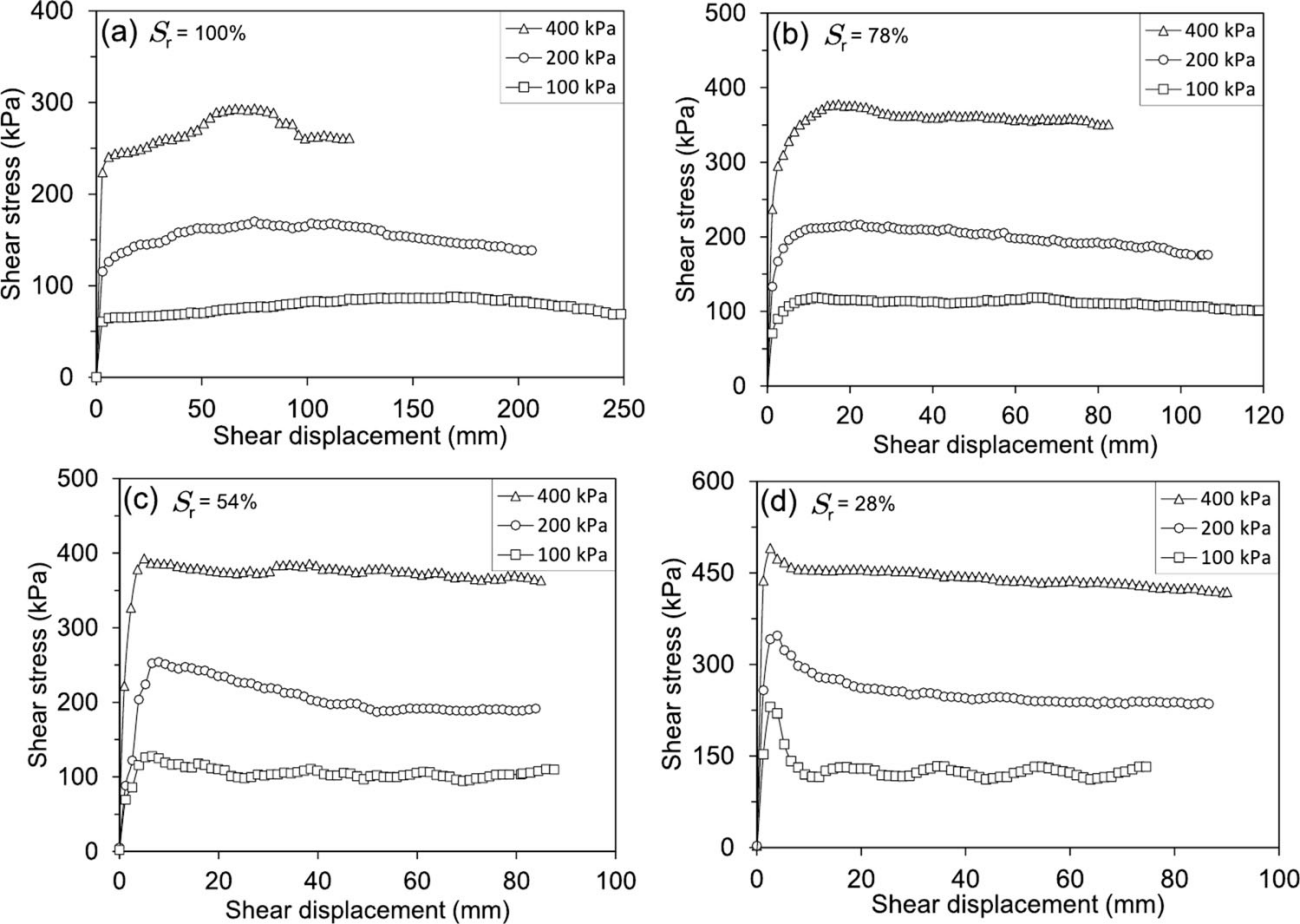
Shear stress-displacement curves of soil samples with (**a**) 100%, (**b**) 78%, (**c**) 54%, and (**d**) 28% degrees of saturation, tested under different effective normal stresses using the RS apparatus

**Fig. 17:**
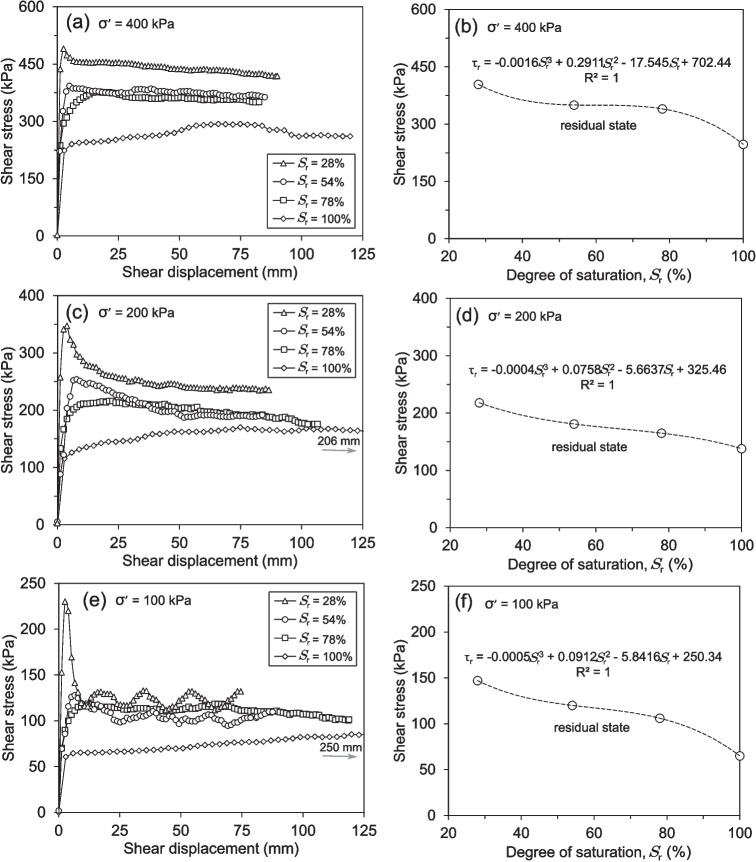
RS test results tested under different effective normal stresses: (**a, c, e**) Shear stress-displacement behaviour of soil specimens with varying initial degrees of saturation. Due to the significant displacement required to achieve residual strength, numerical annotations have been included to indicate the distance necessary to reach the residual value. (**b, d, f**) Variation in residual shear stress as a function of the degree of saturation, illustrating the relationship between saturation levels and residual shear strength

To better interpret the test results, photographs were taken to document the soil samples after shearing. Figure 15a illustrates the shearing gap between the upper and lower shear boxes, highlighting the displacement that developed after four shearing cycles. This visible separation indicates the extent of shear deformation, which is essential for reaching the residual state. Figure 15b presents a detailed view of the upper and lower shear boxes after testing, revealing the shear surface characterised by slickensides. The development of slickensides suggests a polished, low-resistance shear plane due to continuous displacement. These macroscopic features indicate that particle orientation along the shear direction has occurred.

#### Ring shear tests

Figure 16 presents the shear stress-shear displacement behaviour of shear-zone soil samples under different degrees of saturation and effective normal stresses. As shown in Fig. 16a, the fully saturated soil samples exhibited a gradual increase in shear stress, followed by a slow decrease towards the residual state. Under lower effective normal stresses (100 kPa and 200 kPa), the transition from peak to residual shear stress occurs progressively, requiring approximately 250 mm of shear displacement. However, at a higher normal stress of 400 kPa, the residual state is reached within a shorter displacement of about 125 mm, indicating that higher effective normal stresses accelerate the attainment of residual shear strength. The response of partially saturated specimens follows a similar trend but with distinct differences in peak and residual behaviours. As seen in Figs. 16b and c, for degrees of saturation of 78% and 54%, respectively, the peak shear stress increases more rapidly than in the fully saturated samples, and the post-peak reduction is more pronounced, especially for the soil samples with a 54% degree of saturation. The soil samples with the lowest degree of saturation (28%) in Fig. 16d exhibit a notably different response. Shear stresses rise sharply within the first few millimetres of displacement, reaching a pronounced peak within approximately 2 to 3 mm, regardless of the applied normal stresses. A subsequent rapid decline in shear stress follows, leading to a more abrupt transition from peak to residual strength. This behaviour contrasts with the fully saturated specimens, where the reduction in shear stresses is more gradual. The shear displacement needed to attain the residual state decreases with lower degrees of saturation and higher effective normal stresses, underscoring the significant influence of saturation on strain-softening behaviour. Similar shear responses have also been reported by Yang and Vanapalli (2024) for unsaturated fine-grained soils, investigated using a suction-controlled ring shear apparatus.

Similarly, as observed in the RDS tests, Fig. 17 shows the shear stress-shear displacement response of the soil samples tested using the RS apparatus under the same range of degrees of saturation and displacement rates but different effective normal stresses. The influence of the degree of saturation on the shearing behaviour of the soil specimens under 400 kPa is clearly observed in Fig. 17a. Lower degrees of saturation resulted in significantly higher peak and residual shear strengths, indicating a more pronounced strain-softening behaviour. Additionally, as previously discussed, the shear displacement required to reach the residual shear strength increased with higher degrees of saturation. For the specimens under 200 kPa (Fig. 17c) and 100 kPa (Fig. 17e), the differences between peak and residual shear strengths are more pronounced for the soil samples with a 28% degree of saturation, exhibiting a sharp post-peak strength reduction. Conversely, the fully saturated samples displayed a more gradual transition to the residual state, requiring greater shear displacement. The overall trend suggests that lower degrees of saturation contribute to enhanced peak shear strength while increasing saturation leads to a reduction in both peak and residual shear strength. Figure 17b presents the residual shear strength as a function of the degree of saturation for an effective normal stress of 400 kPa. The results demonstrate a gradual reduction in residual shear strength with increasing saturation. However, the most significant decline occurs between the lowest (28%) and highest (100%) degrees of saturation, while the residual values remain relatively stable for intermediate saturation levels (54% and 78%). This trend is also evident for specimens subjected to lower effective normal stresses, as depicted in Figs. 17d and f, where the residual shear strength follows a similar decreasing pattern with increasing saturation.

**Fig. 18:**
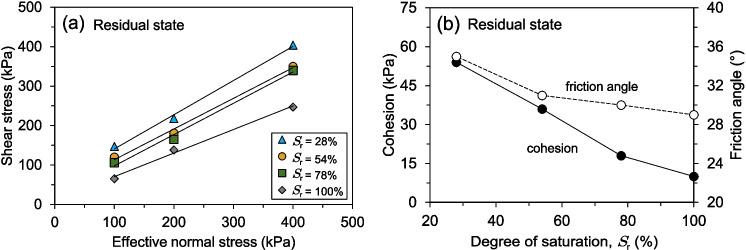
RS tests results: (**a**) Residual shear strength envelopes and (**b**) Variations in cohesion and friction angle for soil samples with differing degrees of saturation

Figure 18a depicts the residual shear strength envelopes obtained under varying degrees of saturation and effective normal stresses. The data indicates that the residual shear strength declines as the degree of saturation increases. However, the most substantial change in residual shear strength occurred between the samples with 100% and 78% degrees of saturation. Further reductions in the degree of saturation did not bring about significant alterations in residual shear strength. Regarding the fluctuations in cohesion and friction angle of the specimens for the residual shear strength under varying degrees of saturation, as shown in Fig. 18b, there is an evident dependence of the residual shear strength parameters on the degree of saturation. Both cohesion and angle of friction decreased as the degree of saturation increased. Notably, the decrease in cohesion is more pronounced than the decrease in the angle of friction. The cohesion demonstrated a practically linear decreasing tendency, while the friction angle after the 78% saturation level followed an almost horizontal path.

To further examine the shear behaviour of the soil samples, additional photographs were taken after testing on the ring shear apparatus. Figure 15c presents the shear-zone soil sample within the shear box, highlighting the twelve evenly spaced ribs or knives. These features play a crucial role in transferring the torsional force to the specimen, facilitating the development of a well-defined shear surface. Figure 15d presents a detailed view of the upper and lower rings, revealing the shear surface formed after shear displacement. Similar to the reversal direct shear tests, the shear surface exhibits particle orientation, as evidenced by the presence of slickensides, indicative of a well-developed shear plane. However, a distinctive feature observed in the ring shear tests is the emergence of outward-spreading circular ridges, which form as a result of prolonged displacement. These ridges reflect the structural adjustments occurring within the shear-zone soil under sustained shearing.

## Discussion

The comprehensive experimental investigation, utilising diverse testing apparatus and conditions, clearly demonstrated that the degree of saturation is a crucial factor influencing both the compressibility and shear strength parameters of the shear-zone soil within the studied slow-moving landslide. The findings indicate that variations in saturation levels lead to significant and irreversible alterations in the soil’s mechanical behaviour, leading to structural degradation and damage over time. Furthermore, the comparative analysis of shear strength parameters, including cohesion and friction angle, as measured using both the direct shear and ring shear devices, revealed a high degree of consistency in the results obtained at both the peak and residual states. This consistency reinforces the reliability of the employed testing methods and further validates the observed trends in soil behaviour under different saturation conditions.

### Variation in compressibility

The consolidation tests, conducted on soil specimens with varying degrees of saturation (28% to 100%), reveal a strong correlation between initial void ratio, water content, and compressibility. Figure 10 clearly illustrates the direct relationship between the initial void ratio and water content and their effect on the compressive behaviour of the soils. This dependence stems from the soil’s shrinkage behaviour during drying. As water content decreases, the soil undergoes volumetric contraction, leading to a reduction in the void ratio. This highlights the significant impact of initial moisture conditions on the soil’s structure and subsequent mechanical response.

Under initial loading, the fully saturated sample exhibits the highest compressibility, with a substantial void ratio reduction. This is expected, as saturated soils, with their pores completely filled with water, are more susceptible to volume change under applied pressures. Conversely, unsaturated samples display progressively lower compressibility with decreasing saturation levels, indicating a stiffening effect from the presence of air within the soil matrix (Behbehani and McCartney 2022; Qin et al. 2023; Sato et al. 2024). This trend underscores the role of water in facilitating soil particle rearrangement under load.

An interesting observation emerges during the unloading and reloading phases. Unsaturated specimens exhibit minimal swelling compared to the fully saturated sample, evidenced by the near-horizontal slopes in Figs. 10b, c, and d. This suggests that the reduced water content and presence of air within the soil structure limits the soil’s ability to rebound upon stress removal. Furthermore, the reloading curves for partially saturated samples closely follow the unloading paths, indicating a linear behaviour in contrast to the non-linear behaviour exhibited during the loading-unloading cycles. However, the fully saturated sample deviates from this trend, displaying a distinct hysteresis loop (Fig. 10a). This discrepancy likely arises from the expulsion and subsequent re-entry of water within the soil pores during unloading and reloading, leading to irreversible structural changes.

The calculated compression index $C_c$ decreased with decreasing degree of saturation, reflecting the reduced compressibility of the soil (Fig. 11) (Behbehani and McCartney 2022; Qin et al. 2023). The most substantial drop occurred between the 78% and 54% saturation levels, suggesting a potential critical threshold. Above this threshold, the soil’s water content appears to be the dominant factor, while below it, the air content and its influence on the soil structure likely become more crucial in governing the compression behaviour. Similarly, the swelling index $C_s$, which quantifies the soil’s ability to swell, also decreased with decreasing saturation (Fig. 11). The most pronounced change was observed between the fully saturated and 78% saturated samples. As the saturation decreases, the air trapped within the soil pores hinders water ingress, thereby limiting the soil’s swelling potential. The near-identical swelling index values between the 78% and 54% saturation levels may indicate a range where the effects of air entrapment become significant but relatively stable.

### Variation in shear strength parameters

Combining the results from both Reversal Direct Shear (RDS) and Ring Shear (RS) tests provides a comprehensive understanding of how the degree of saturation influences the peak and residual shear strength of the studied soil. Across both testing methods, a consistent trend emerges: increasing saturation leads to a reduction in both peak and residual shear strength (Fig. 13b, d, f; Figs. 17b, d, f). This phenomenon is primarily attributed to the lubricating effect of water within the soil pores. As saturation increases, pore water pressure rises, reducing inter-particle contact forces and weakening the soil’s resistance to shearing. This effect is particularly evident in fully saturated samples tested under 100 kPa and 200 kPa effective normal stresses in the RS device, where a gradual decline in shear stress is observed before reaching residual conditions (Fig. 16a).

The relationship between saturation and shear displacement further highlights the soil’s mechanical response. Soil samples with the lowest degree of saturation (28%), regardless of the testing method, exhibit a rapid increase in shear stress, reaching peak strength at very low displacements (Figs. 12d, 16d). This suggests a more brittle response in drier soils, with limited strain-softening behaviour. In contrast, saturated samples, particularly in the RS tests, require significantly larger shear displacements to mobilise peak strength, indicating a more ductile response (Fig. 16a). This difference in behaviour underscores the critical role of degree of saturation in dictating the soil’s mechanical response to shearing. The trend of decreasing residual shear strength with increasing saturation remains consistent across both RDS and RS tests (Figs. 13b, d, f; Figs. 17b, d, f). This highlights that even after significant particle rearrangement during shearing, the presence of water continues to influence the soil’s inherent shear resistance. Interestingly, the most significant drop in residual strength occurs between 100% and 78% degrees of saturation (Fig. 18a), suggesting a threshold beyond which further reductions in water content have a less pronounced effect on residual shear strength.

The influence of effective normal stress further complicates this relationship. As shown in Figs. 13a, c, and e, the overall shear strength values decrease with decreasing effective normal stress, regardless of the degree of saturation. However, the effect of saturation on peak and residual shear strength becomes more pronounced at lower normal stresses. For example, the difference between peak and residual shear strength is more significant for the 28% degree of saturation sample under 50 kPa normal stress compared to higher normal stresses (Fig. 13e). This suggests that the mechanical response of the soil is highly dependent on both saturation and the applied stress conditions. At higher normal stresses, the influence of the degree of saturation on shear strength is somewhat mitigated, as the increased inter-particle contact forces partially counteract the weakening effect of pore water pressure. The strain-softening behaviour of the soil is another critical aspect. In the RS tests, the transition from peak to residual shear stress at saturated conditions occurs more rapidly at higher effective normal stresses, requiring approximately 125 mm of shear displacement at 400 kPa compared to 250 mm at 100 kPa (Fig. 16a). This indicates that higher normal stresses accelerate the attainment of residual shear strength, likely due to increased particle crushing and rearrangement under greater loads. Conversely, lower degrees of saturation result in a more abrupt transition from peak to residual strength, as seen in the samples with a 28% degree of saturation (Fig. 16d). This behaviour contrasts with the gradual strain-softening observed in fully saturated samples, further emphasising the role of saturation in modulating the soil’s post-peak response.

Analysing the changes in cohesion and friction angle with varying saturation levels provides valuable insights into the underlying mechanisms (Figs. 14b, d, 18b). While both parameters generally decrease with increasing degrees of saturation, reflecting the combined influence of pore pressure and reduced inter-particle bonding, some key observations can be made. The decrease in cohesion is significantly more pronounced than that of the friction angle, particularly at higher saturation levels. This suggests that the loss of apparent cohesion due to reduced capillary forces plays a dominant role in the overall weakening of the shear-zone soil. The near-linear decrease in residual cohesion with increasing saturation further supports this observation (Fig. 18b). However, the friction angle, which represents the resistance to sliding along particle surfaces, exhibits a more gradual decline, indicating that inter-particle friction is less sensitive to changes in saturation compared to cohesion. Although both cohesion and friction angle generally decrease with increasing degrees of saturation, this may not solely be due to the influence of pore pressure and reduced inter-particle bonding. The friction angle could also be impacted by factors such as particle rearrangement, particle shape, and changes in the soil’s microstructure (Kang et al. 2022c; Su et al. 2023; Miao et al. 2024). For instance, the more pronounced peak strength observed in samples with lower saturation levels (Fig. 12d, 16d) may be attributed to the preservation of a more stable particle structure, which is disrupted as the degree of saturation increases. Similarly, the decrease in cohesion might not be entirely attributed to the loss of apparent cohesion due to reduced capillary forces. Other physicochemical interactions, such as changes in surface charges and the formation of new mineral phases, could also contribute to the observed reductions in cohesion (Bjerrum 1967; Leroueil 2001; Lacroix et al. 2020; Su et al. 2023). These interactions may alter the soil’s internal bonding mechanisms, further complicating the relationship between saturation and shear strength.

The shear behaviour of soil samples subjected to prolonged and repeated shearing cycles demonstrates notable structural adjustments within the shear zone, directly influencing the variation in shear strength parameters. As observed in the tests conducted at a degree of saturation of 28%, these adjustments include the formation of shearing gaps, slickensides, and outward-spreading ridges, which contribute to changes in both peak and residual shear strength. As shown in Fig. 15b, the visible separation between the upper and lower shear boxes after four cycles highlights the development of slickensides on the shear surfaces, indicating a progressive reorientation of soil particles. This reorganisation leads to the formation of a polished, low-resistance shear plane, reducing shear strength and promoting strain localisation. Similarly, Fig. 15d illustrates the emergence of outward-spreading circular ridges in the ring shear tests, a distinctive feature resulting from prolonged displacement. These structural modifications suggest continuous particle realignment and microstructural reorganisation, ultimately altering the residual shear strength of the soil. The findings from both testing methods emphasise the significant role of shear-induced fabric changes in governing shear strength parameters, particularly in relation to the long-term stability of slow-moving landslides.

## Conclusions

This research work investigated the implications of varying degrees of saturation on the mechanical behaviour of a slow-moving landslide in the TGR area of China. A series of laboratory tests were conducted on shear-zone soil specimens under different saturation levels. Consolidation tests were used to determine the compressibility characteristics of the soil, while reversal direct shear (RDS) tests and ring-shear (RS) tests were employed to analyse the peak and residual shear strength and the effects of the degree of saturation on the specimens. The following conclusions can be summarised:

1. The variation in compressibility emphasises the profound influence of saturation and shrinkage behaviour on the mechanical response of the studied soil. While fully saturated samples exhibit higher compressibility and hysteresis, unsaturated samples demonstrate lower compressibility and a more elastic response. These observations further support the notion that capillary action, air entrapment, and changes in interparticle forces within the soil play a crucial role in influencing the soil’s swelling and shrinking behaviour.
2. The relationship between soil compressibility and saturation follows a consistent trend across different soil types. Higher saturation levels generally lead to increased compressibility, while lower saturation reduces both the compression and swelling indices. The most significant changes in these indices typically occur at moderate saturation levels, highlighting the importance of considering a full range of saturation conditions when assessing soil consolidation behaviour.
3. Both RDS and RS tests consistently demonstrate that increasing saturation reduces both peak and residual shear strength. This behaviour is likely attributable to the combined effects of increased pore water pressure and diminished inter-particle bonding. While the loss of apparent cohesion appears to play a dominant role, further research is needed to fully elucidate the complex interplay of factors influencing shear strength behaviour under varying degrees of saturation.
4. Shear strength parameters, including cohesion and friction angle, exhibit predictable variations with changes in saturation. As saturation increases, cohesion tends to decrease significantly, while the friction angle follows a more gradual decline. Additionally, the shear displacement required to reach residual strength increases with higher saturation levels, while unsaturated soils experience more abrupt reductions in shear stress. These findings reinforce the broader understanding that saturation plays a critical role in determining the mechanical stability of shear-zone soils in landslide-prone areas.
5. While the current study primarily focuses on laboratory experiments, we acknowledge the importance of integrating field data to provide a more comprehensive analysis. Future research will aim to collect field measurements, such as displacement rates, moisture content variations, and pore pressure data, to further investigate the failure mechanisms of slow-moving landslides in the Three Gorges Reservoir area.

In short, these findings highlight the importance of considering a wider range of degrees of saturation when analysing and predicting the deformation behaviour and shear strength parameters of shear-zone soils in slow-moving landslides. This comprehensive understanding of the impact of varying saturation levels is essential for a better understanding of the potential failure mechanisms involved in such landslide systems.
